# Ketogenic Diet in Schizophrenia: A Critical Review and Framework for Future Implementation

**DOI:** 10.1192/bjo.2026.11115

**Published:** 2026-06-30

**Authors:** Aarv Paul, Gurpreet Kaler

**Affiliations:** 1 University of Sheffield, Sheffield, United Kingdom; 2 Nottinghamshire Healthcare NHS Foundation Trust, Retford, United Kingdom

## Abstract

**Aims:**

Current antipsychotic treatments in schizophrenia inadequately address negative and cognitive symptoms and are associated with significant metabolic side effects, contributing to poor adherence and relapse. This review evaluates and synthesises current evidence on the ketogenic diet in schizophrenia and identifies methodological limitations to inform future research.

**Methods:**

A systematic search was conducted across PubMed, MEDLINE, Embase, PsycINFO, and Scopus using terms related to “ketogenic diet” and “schizophrenia”. Primary studies involving participants with schizophrenia receiving a ketogenic diet were included; animal studies, secondary analyses, non-ketogenic or exogenous ketone interventions, and non-English publications were excluded. Of 1177 records identified, 11 studies met the inclusion criteria and were appraised using Joanna Briggs Institute tools.

**Results:**

Across 11 studies involving 38 participants, with follow-up ranging from 2 weeks to 12 years, ketogenic diet interventions were associated with improvements in psychotic and mood symptoms, often observed within weeks. Several studies also reported improvements in metabolic markers and physical well-being and reduced antipsychotic use, including complete withdrawal in some participants. Greater adherence was reported when participants received structured support. Symptom relapse was reported following diet discontinuation, suggesting possible diet-dependence. Reported adverse effects were generally mild and transient.

**Conclusion:**

Preliminary evidence suggests potential mental and metabolic benefits of the ketogenic diet in schizophrenia; however, current findings are limited by small sample sizes, reliance on case reports and pilot designs, and inconsistent adherence. Future large-scale, controlled trials should use standardised symptom measures and systematic reporting of psychiatric and metabolic outcomes to establish efficacy, safety, and comparability.

The authors have not received any financial sponsorship.

